# Occupational symptoms due to exposure to volatile organic compounds among female Vietnamese nail salon workers in Danang city

**DOI:** 10.1002/1348-9585.12160

**Published:** 2020-09-18

**Authors:** Huan M. Tran, Hanh T. M. Bui, Somkiat Thoumsang, Ngoc T. B. Ngo, Nhan P. T. Nguyen, Hai T. M. Nguyen, Son M. Nguyen, Kunio Hara, Supat Wangwongwatana

**Affiliations:** ^1^ Faculty of Public Health Thammasat University Rangsit Thailand; ^2^ Faculty of Public Health Da Nang University of Medical Technology and Pharmacy Da Nang Viet Nam; ^3^ Department of Quarantine Service Danang Center for Disease Control Da Nang Viet Nam; ^4^ Institute for Community Health Research Hue University of Medicine and Pharmacy Hue University Hue Viet Nam; ^5^ Faculty of Pharmacy Da Nang University of Medical Technology and Pharmacy Da Nang Viet Nam; ^6^ Faculty of Odonto‐Stomatology Da Nang University of Medical Technology and Pharmacy Da Nang Viet Nam; ^7^ Department of Safety and Health Management University of Occupational and Environmental Health Kitakyushu Japan

**Keywords:** nail workers, occupational symptoms, Vietnam, volatile organic compounds

## Abstract

**Objectives:**

Nail workers are exposed to many hazardous chemicals. Despite many warnings about health problems among nail workers in other countries, data concerning exposure to chemical hazards among nail workers is still limited in Vietnam. In this study, we aimed to identify exposure to volatile organic compounds and their relationship with occupational symptoms among Vietnamese female nail salon workers.

**Methods:**

A cross‐sectional study was conducted in Danang, Vietnam, from January 2019 to September 2019. Total 42 personal passive samplers were collected to evaluate 12 substances from 21 nail workers (15 salons) twice a week. We chose one representative worker from each of the nine salons with less than six workers and two representative workers from each of the six salons with over five workers for personal sampling based on the principle of similar exposure groups. We interviewed a total of 100 nail workers in 15 salons and 100 office workers in offices adjacent to the salons to compare occupational symptoms among them.

**Results:**

The commonly detected compounds in nail salons were acetone (97.6%), butyl acetate (83.3%), and ethyl acetate and ethyl methacrylate (78%). The concentration of total target VOCs was related to the number of serviced customers, the concentration of CO_2_, and general ventilation used. The subjective symptoms were significantly higher for the nail workers than for the comparison subjects, that is, headache, nausea, nose irritation, skin irritation, shortness of breath, and confusion. Among 100 nail workers, nose irritation was significantly higher for nail workers who were exposed to acetone at levels exceeding the Vietnam occupational exposure limit (VOEL) adjusted with the Brief‐Scala model.

**Conclusions:**

Exposure to VOCs such as acetone in nail salons results in occupational symptoms among workers.

## 1 INTRODUCTION

Nail workers are exposed to a variety of hazardous chemicals, including methyl methacrylate acetone, toluene, and butyl acetate.^1^ Compared to the general population, nail workers suffer from both short‐term health problems, such as from damage to the central nervous system and respiratory issues as well as long‐term effects including cancer and non‐cancerous diseases.^1^, ^2^, ^3^, ^4^ Almost 90% of the 1000 chemicals in nail products have not been assessed for safety.^5^ The concentration of some chemicals in the nail shops in Vietnam exceeds the occupational limits of some countries such as Korea,^1^ Britain,^3^ and the United States .^5^ Particularly, many studies found more than 25 substances in nail polish removers, nail hardeners, artificial nails, and disinfectants that can affect human health.^2^ In addition, some nail shops use personal care products containing banned chemical substances, especially methyl methacrylate.^6^ Vietnamese nail workers play a key role in the nail industry in developed countries. The booming nail industry has resulted in the prevalence of Vietnamese immigrant workers increasing from 10% to 59% in the last two decades in the United States.^7^ Health issues among Vietnamese nail workers are one of the concerns of occupational health officers in the United States and Europe.^4^, ^5^, ^8^, ^9^, ^10^ Quach conducted many studies and interventions among Vietnamese workers to promote their health and safety in nail salons in the United States.^11^, ^12^ For example, they improved the knowledge and behavior of nail salon workers to reduce workplace chemical exposure.^12^

Public health officers have raised concerns about the impact of nail care products on the health of nail workers because of the diverse toxic substances affecting the respiratory system.^1^, ^4^, ^5^, ^6^ Some of these substances have been associated with acute effects such as headache; vomiting; and skin, eye, and respiratory irritation.^2^, ^3^, ^4^, ^13^, ^14^ Moreover, the route, frequency, concentration, and period of exposure to some nail product substances have a close relationship with the incidence of chronic diseases such as cancer and reproductive and respiratory system disorders.^2^ Tsigonia et al found that nail workers tend to inhale volatile organic compounds (VOCs) despite good ventilation.^15^ Goldin et al showed that insufficient ventilation results in high CO_2_ and VOCs exposure.^16^ Quach et al conducted community‐based participatory research to recruit nail salon workers and record the occupational symptoms in this group.^5^ Harris et al found a higher proportion of respiratory symptoms among nail workers than among the office staff.^3^ Park et al suggested that local exhaust ventilation can be used to protect nail workers.^1^ White et al emphasized the need for researchers to find the association between exposure to chemicals and health outcomes.^17^ Shendell et al proposed that nail workers undergo comprehensive training in nail product use and that additional research should be conducted to determine the extent of exposure.^4^ Although there have been some cross‐sectional studies, data on the association between chemical exposure and occupational symptoms among nail salon workers remain limited.

As the demand for nail care services has increased in Vietnam, nail care workers do not need to immigrate to the United States and Europe for occupational success.^3^, ^5^ A large number of Vietnamese nail workers have started businesses in their own countries to improve their lives or to wait for approval to reside in foreign countries.^18^ Despite many warnings about health problems among nail workers in other countries, the data concerning exposure to chemical hazards among nail workers is still limited in Vietnam. Therefore, there is a need for research concerning chemical exposure and occupational symptoms among female nail workers in Vietnam. In this study, our objective was to identify the related factors of VOCs that place Vietnamese nail salon workers at risk of occupational symptoms.

## 2 METHODS

We conducted a cross‐sectional study in Danang, Vietnam, from January 2019 to September 2019. According to our calculations, the number of study participants needed to detect the symptoms in the exposed nail salon workers was 83. Assuming sample loss of approximately 10%, the required sample size was 92. Overall, 100 nail workers participated in this study. See formula #1.

(1) $$n=Z_{1‐\frac{\alpha }{2}}^{2}\frac{p(1‐p)}{d^{2}}$$

Where:

p = Using proportion of detecting at least one symptom among nail workers (*P* = .313)^5^

n = Sample size of nail workers.

d = Relative error given, d = 0.1

α = 0.05, $Z_{1‐\frac{\alpha }{2}}^{2}$=1.96^2^.

### 2.1 Recruitment of Subjects

#### 2.1.1 Nail worker group

To recruit subjects exposed to occupational toxic chemicals, we selected potential nail salons in Danang, Vietnam, from the list of salons maintained by a local officer. We also sent our research assistants to identify additional salons not on the list. A total of 83 nail salons were identified by the local officer and our assistants.

After the 83 nail salons were identified, the research assistants visited each establishment and provided an introductory letter from Danang University of Medical Technology and Pharmacy, information related to purpose and methods of the study, and consent forms. Only eight salons agreed to participate in this study. This is less than 10% of the identified nail salons. To supplement our sample of nail salons, we relied on salon owners to further recruit other potential establishments. This “snowball effect” led to the addition of seven nail salons. As a result, we selected 15 nail salons with 100 nail workers.

All our exposed subjects were Vietnamese female nail salon workers. All these workers signed the consent form for voluntary participation. The exclusion criteria were pregnancy, inability to communicate in Vietnamese, and less than 1 month of employment in the nail salon industry.

#### 2.1.2 Office worker group

An office worker group was selected to better understand the complaints related to occupational symptoms made by nail shop workers who were not directly exposed to the same occupational toxic chemicals. These individuals mainly worked in a seated position without handling organic chemicals. The office workers such as bankers, sellers, managers, and others working on the same street as the salons, were age matched with the nail workers. The same inclusion and exclusion criteria were used for the comparison groups as the nail salon worker group.

#### 2.1.3 Measurements of the nail worker and office worker groups

An interview guide was used to conduct face‐to‐face interviews with nail workers during their non‐working hours. The following data were recorded: age; marital status; alcohol consumption; smoking status; physical activity level; ethnicity; working hours per week; work experience; use of personal protective equipment; periodic health examinations; self‐defined health; and occupational symptoms experienced in the previous month, and these symptoms disappeared while they were away from the workplace for more than 1 day, including headache, nose irritation, skin irritation, throat irritation, cough, nausea, chest tightness, shortness of breath, and confusion. The same questionnaire was used to interview the office workers in the comparison group.

#### 2.1.4 Measurements of the salon

We measured the salon length, width, and height to calculate the volume of the salon. The average temperature, humidity, ventilation airflow, and CO_2_ were measured during working hours four times daily: at opening time in the morning, at 11 AM, at 1 PM, and at closing time. Measurements were taken using the Q‐Trak Indoor Air Quality Monitor 7575 (calibrated in 1/2019). In addition, we inspected the natural (open windows or doors) and engineered (local or exhaust) ventilation systems and any adjacent gas station, which can affect the VOCs level in the nail salons. One assistant in each salon counted the number of customers on the day of measurement and the number of customers serviced by the workers who were equipped with the monitor.

We asked the workers who participated in the study to wear a personal air‐monitoring (3M 3500 Organic Vapor Monitor) device during their work shift. We chose one representative worker from salons with less than six workers (nine salons) and two representative workers from salons with more than five workers (six salons) based on the owners' suggestion of similar exposure groups (SEGs) because these salons are large and difficult to represent using a single sample. The total samplers were calculated:

$$\left[9\left(\text{small salons}\right)\times 1\left(\text{sampler}\right)+6\left(\text{large salons}\right)\times 2\left(\text{samplers}\right)\right]\times 2\left(\text{days}\right)=42\left(\text{samplers}\right)$$

Furthermore, the assumption in assigning SEG was that air sampling performed on any member of the SEG applied for all the SEG members.^19^

We measured the personal concentration twice weekly, on one weekday and on one weekend day; the number of customers before sampling on weekdays and weekends was self‐reported by owners (not observed by the investigators).

The sampler was clipped to the workers' collar of the breathing zone. We conducted approximately full shift sampling. Seven field blanks were used in seven sampling days to control for any contaminants the samplers may be exposed during transport and deployment procedures that are not actually representative of the sampling site. The field blank was not deployed in the environment. The field blank was taken while going out to set up samplers. The field blank was also brought back, packaged, and sent for analyses.

#### 2.1.5 Air sample handling and transport

As acetone in the passive sampler can evaporate in high humidity and temperature, the passive samplers were stored at 4°C with dry ice during transportation from the field to Thammasat Laboratory and we waited for up to 1 month until laboratory analysis according to the suggestion of 3M.

#### 2.1.6 Analysis of air samples

Laboratory analysis was conducted by a specialist in the GC‐FID of the Faculty of Public Health of Thammasat University, Thailand. We chose 12 common compounds based on the review and recommendation of the 3M company for analysis, including ethanol, acetone, methyl ethyl ketone, benzene, toluene, ethyl benzene, xylene, ethyl acetate, n‐butyl acetate, vinyl acetate, ethyl methacrylate, and methyl methacrylate. We used 2 mL of CS_2_ solvent desorption followed by gas chromatography with a flame ionization detector to determine the chemicals on the charcoal absorbent. The NIOSH method was used to analyze passive samplers after desorption.^20^, ^21^, ^22^, ^23^, ^24^, ^25^, ^26^, ^27^ This method is used for detecting VOCs in passive samplers. The concentration of chemicals was analyzed based on the following formula on the recommendation of 3M. If the field blank has contaminants, the concentration of chemicals will be minus to the concentration in the field blank.

$$C\left(ppm\right)=\frac{W\left(micrograms\right)\times B}{r\times t\left(minutes\right)};B=\frac{1000\times 24.45}{samplingrate\times molecularweight}$$

where C is the contaminant identity (ppm), t is the sampling time in minutes, B is the calculation constant, r is the recovery coefficient, and W is the contaminant weight (in grams).

The Vietnamese National Technical Regulation has proposed the Brief‐Scala Model to adjust the exposure limit for workers working more than 8 h/day or 40 h/week working in the vicinity of hazardous chemicals.^28^ The workers would be classified to exceeding occupational exposure limit if their salon had any sampler exceeding the adjusted Vietnamese occupational exposure limit (VOEL). See formula #2.

(2) $$RF=\frac{40}{h}\times \frac{168‐h}{128};\mathrm{adjusted}\mathrm{VOEL}=\mathrm{VOEL}\times \mathrm{RF}$$

RF = reduction factor.

h = average hours per week.

VOEL = Vietnam occupational exposure limit.

Quality assurance procedures included field and solvent blanks to check for contamination, using regular calibration with certified standards. Field blank was conducted for each sampling day. The target VOCs were calculated as the sum of concentrations of detected VOCs.

### 2.2 Data analysis

The data were entered by Epidata 3.1 onto two separate files. We checked for errors by comparing the two files and the original questionnaires. The data were analyzed using RStudio Statistical Software Version 1.1.463 Mac OS X 10_12_6.

We used the χ^2^ test and t‐test to compare various characteristics of the nail workers and the office workers. Multiple logistic regression analysis was used to analyze the results of health symptoms among nail workers who either exceeded or did not exceed the VOEL for acetone. Odds ratios (ORs) were adjusted for the potential confounding effects of age, smoking, alcohol consumption, exercise frequency, work experience, and marital status. The same methods were used to compare the results between office workers and nail workers.

We used the Akaike information criterion to check the distribution of the concentration of target VOCs and acetone before placing them in a Gamma family attached to the log‐link function of the generalized linear model. We used the Bayesian Model Averaging package for choosing the predicted factors for the concentration of target VOCs and acetone because of the small sample size with other chemicals.

## 3 RESULTS

The 15 salons were measured over 2 days of the week, and 42 measurements were taken in the salons. The basic characteristics of the working environment in nail salons at the time of sampling are summarized in Table 1. The average time of personal air sampling is 8.28 (equal to 92% of the working time). The average temperature, relative humidity, ventilation, CO_2_ concentration, and volume of salons were 33.0°C, 64.3%, 0.37 m/s, 940 ppm, and 120 m^3^, respectively. Each salon had an average of 4.8 workers. The average volume of salons was approximately 120 m^3^.

**Table 1:** Characteristics of nail salons

Characteristics	Mean ± SD
Salons	
Number of workers (n = 30)	4.77 ± 3.31
Number of customers (n = 30)	11.1 ± 9.52
Number of customers serviced by sampler workers (n = 42)	2.85 ± 2.11
Sampling time, h (n = 42) (Mean (range))	8.28 (4.2‐10.8)
Temperature ^o^C (n = 30)	33.02 ± 14.11
Relative humidity, percentage (n = 30)	64.32 ± 33.80
Air velocity, m/s (n = 30)	0.37 ± 0.15
Volume, m^3^ (n = 15)	119.58 ± 70.43
CO_2_ exposure, ppm (n = 30)	940.29 ± 504.73

Abbreviation: SD, Standard deviation; n = 15 (number of salons); n = 42 (number of samplers); n = 30 (frequency of measurement of the characteristics) (15 salons ×2 times).

A total of 12 substances were analyzed and detected from 42 personal samples of the nail shop workers: one alcohol (ethanol), five esters (ethyl acetate, butyl acetate, vinyl acetate, methyl methacrylate, and ethyl methacrylate), two ketones (acetone and methyl ethyl ketone), and four aromatic hydrocarbons (benzene, toluene, ethylbenzene, and xylene). The most commonly reported compound with the highest concentration in the nail salons was acetone, detected in 97.6% of the samples. The concentrations of target VOCs and individual VOCs, such as acetone, were approximately Gamma distributed. The VOEL of ethanol, acetone, benzene, vinyl acetate, methyl ethyl ketone, n‐butyl alcohol, methyl methacrylate, toluene, butyl acetate, and xylene were 530, 85, 1.6, 2.8, 50, 50, 13, 40, 100, and 23 ppm, respectively (Table 2).

**Table 2:** Airborne concentration of chemicals in nail salons (ppm) (n = 42)

Chemicals	Det (%)	AM	SD	GM	GSD	Min	Max	VOEL
Ethanol	13 (31.0)	4.28	5.67	1.83	4.38	0.24	20.36	530
Acetone	41 (97.6)	8.92	10.41	4.26	3.75	0.45	34.80	85
Methyl ethyl ketone	33 (78.6)	0.26	0.09	0.25	1.42	0.14	0.49	50
Benzene	9 (21.4)	0.03	0.03	0.02	2.09	0.01	0.1	1.6
Toluene	27 (64.3)	0.09	0.12	0.06	2.38	0.01	0.63	40
Ethyl benzene	4 (9.5)	0.06	0.01	0.06	1.18	0.05	0.07	
Xylene	11 (26.2)	0.05	0.03	0.04	1.78	0.02	0.10	23
Ethyl acetate	31 (73.8)	0.22	0.11	0.20	1.54	0.1	0.49	
n‐butyl acetate	35 (83.3)	0.18	0.10	0.15	1.84	0.05	0.42	100
Vinyl acetate	12 (28.6)	0.14	0.1	0.10	1.78	0.06	0.4	2.8
Ethyl methacrylate	31 (73.8)	1.32	2.01	0.51	3.22	0.1	3.19	
Methyl methacrylate	21 (50.0)	0.91	1.57	0.32	4.01	0.08	5.69	13
Target VOCs	42 (100)	11.63	10.95	7.40	2.91	0.94	36.64	

Abbreviation: Det (%), Detected proportion of compounds in samplers; AM, Arithmetic mean; SD, Standard deviation; GM, Geometric mean; GSD, Geometric standard deviation; Min, Minimum concentration; Max, Maximum concentration; VOEL, Vietnamese occupational exposure limit; VOCs, Volatile organic compounds.

Based on Bayesian Model Averaging, the number of serviced customers, and the concentration of CO_2_ appears in all models to predict the concentration of target VOCs followed by general ventilation. Similarly, the concentration of acetone can be predicted based on the number of serviced customers, the concentration of CO_2_, the air conditioners used, and artificial service (Table 3).

**Table 3:** Multi‐gamma regression for predicting the concentration of TVOCs and acetone (ppm)

Factors	Target VOCs			Acetone		
	β	SE	P‐value	β	SE	*P*‐value
No. of customers in the salon				1.05	1.01	<.001
No. of serviced customers	1.19	1.05	<0.001	1.49	1.05	<.001
Concentration of CO_2_	1.001	1.002	<0.001	1.001	1.001	<.001
General ventilation	1.44	2.00	*NS			
Air conditioner used				0.25	1.45	<.001
Volume of salon	1.002	1.002	*NS			

Abbreviation: VOCs, Volatile organic compounds; *β,* Regression coefficient; SE, Standard error; *NS, Not significant.

There was no significant difference between the mean age of nail workers (23.97) and the comparison group (24.48). Sixty‐nine percent of the nail workers and 68% of the office workers were married. The mean working career was 1.9 years. In terms of mean weekly work hours, nail workers spent nearly 58 hours in the workplace while office workers spent only 42 hours in the workplace. Most of the nail workers (98%) and office workers (99%) did not smoke or drink alcohol frequently. The proportion of office workers undergoing periodic health examinations was 7% higher than that of the nail workers. Eighty‐seven percent of the nail workers and 94% of the office workers self‐reported being in good health. Nine percent of the nail workers exceeded the VOEL after the Brief‐Scala model adjustment (Table 4).

**Table 4:** Comparison of characteristics between nail salon workers and office worker group (n = 200)

Characteristics (% or Mean ± SD)	Nail salon workers (n = 100)	Office worker group (n = 100)	*P*‐value
Marital status	69	68	NS*
Regular exercise	22	38	<.05
Kinh ethnic group	99	94	NS*
Smoking status	2	1	NS*
Frequent alcohol intake	4	2	NS*
Periodic health examination	20	35	<.05
Good health self‐defined	87	94	NS*
Exceeding adjusted VOEL^a^	9	—	—
Age	23.97 ± 5.65	24.48 ± 2.40	NS*
Working experience	1.87 ± 4.26	2.77 ± 2.08	NS*
Average Working hours a week	58.18 ± 22.63	42.27 ± 8.29	<.01
Number of working hours per day	8.98 ± 2.86	7.89 ± 1.27	<.01
Number of working days per week	6.3 ± 0.88	5.46 ± 0.87	<.01

Abbreviation: NS*, Not significantly.

^a^ adjusted VOEL after the Brief–Scala model adjustment.

Table 5 compares the occupational symptoms between 100 nail workers and 100 office workers. The most frequently reported symptoms among nail workers were headaches (49%), followed by nose irritation (28%) and confusion (19%). In the comparison group, the most frequently complained symptoms were headaches (36%) and nose irritation (21%). The logistic regression analysis found that ORs for subjective symptoms were significantly (*P* < .05) higher for nail workers vs office workers for headache (OR, 3.29; confidence interval [CI], 1.46 to 7.37), nausea (OR, 5.56; CI, 1.14 to 27.12), nose irritation (OR, 2.65; CI, 1.09 to 6.45), skin irritation (OR, 3.53; CI, 1.19 to 10.43), breath shortness (OR, 9.92, CI 2.02 to 48.62), and confusion (OR, 4.94, CI 1.67 to 14.56). After adjusting for working hours from the Brief‐Scala model, nail workers exceeded the Vietnam exposure limit of acetone and showed a higher risk of nose irritation (OR, 32.9; CI, 4.8 to 224.58) compared to office worker group.

**Table 5:** Occupational symptoms of nail salon workers, office workers and nail salon workers exceeding Vietnam occupational exposure limit of acetone

	Symptoms n (%)		Crude OR	Adjusted OR	*P*‐value
	No	Yes			
	Headache				
Office workers	64 (64)	36 (36)	1	1	
Nail workers	51 (51)	49 (49)	1.71 (0.97,3.01)	3.29 (1.46,7.37)	<.01
Exceeding VOEL of acetone	4 (44.4)	5 (55.6)	2.22 (0.56,8.8)	4.53 (0.91,22.67)	*NS
	Nausea				
Office workers	96 (96)	4 (4)	1	1	
Nail workers	89 (89)	11 (11)	2.97 (0.91,9.65)	5.56 (1.14,27.12)	<.05
Exceeding VOEL of acetone	8 (88.9)	1 (11.1)	3 (0.3,30.13)	6.09 (0.38,96.68)	*NS
	Nose irritation				
Office workers	79 (79)	21 (21)	1	1	
Nail workers	72 (72)	28 (28)	1.46 (0.46,2.8)	2.65 (1.09,6.45)	<.05
Exceeding VOEL of acetone	2 (22.2)	7 (77.8)	13.17 (2.6,68.1)	32.9 (4.8,224.58)	<.01
	Throat irritation				
Office workers	92 (92)	8 (8)			
Nail workers	92 (92)	8 (8)	1 (0.36,2.78)	1.33 (0.37,4.78)	*NS
Exceeding VOEL of acetone	8 (88.9)	1 (11.1)	1.44 (0.16,12.98)	2.05 (0.15,28.9)	*NS
	Skin irritation				
Office workers	87 (87)	13 (13)	1	1	
Nail workers	73 (73)	17 (17)	1.37 (0.63,3)	3.53 (1.19,10.43)	<.05
Exceeding VOEL of acetone	8 (88.9)	1 (11.1)	0.84 (0.1,7.25)	1.87 (0.16,21.99)	*NS
	Cough				
Office workers	89 (89)	11 (11)	1	1	
Nail workers	90 (90)	10 (10)	0.9 (0.36,2.22)	1.07 (0.36,3.22)	*NS
Exceeding VOEL of acetone	8 (88.9)	1 (11.1)	1.01 (0.12,8.87)	1.78 (0.14,22.4)	*NS
	Breath Shortness				
Office workers	98 (98)	2 (2)	1	1	
Nail workers	86 (86)	14 (14)	1.98 (1.76,36.09)	9.92 (2.02,48.62)	<.01
Exceeding VOEL of acetone	7 (77.8)	2 (22.2)	14 (1.71,114.85)	27.95 (2.4,321.4)	<.01
	Confusion				
Office workers	94 (94)	6 (6)	1	1	
Nail workers	81 (81)	19 (19)	3.67 (1.4,9.64)	4.94 (1.67,14.56)	<.01
Exceeding VOEL of acetone	6 (66.7)	3 (33.3)	7.83 (1.56,39.31)	17.48 (2.5,123.6)	<.01
	Chest tightness				
Office workers	97 (97)	3 (3)	1	1	
Nail workers	86 (94.5)	6 (6)	2.06 (0.5,8.49)	2.74 (0.55, 13.63)	*NS
Exceeding VOEL of acetone	8 (88.9)	1 (11.1)	4.04 (0.38,43.46)	6.81 (0.36,128.0)	*NS

Abbreviation: OR, Odd ratio; *NS, Not significant.

Logistic regression adjusted for marital status, regular exercise, ethnicity, smoking status, frequent alcohol intake, undergoing periodic health examination, age, and average working hours per week.

## 4 DISCUSSION

The most common and highest concentration compounds in the nail salons were acetone, which was detected in 97.6% of the 42 samples. The most frequently reported symptoms by nail workers were headaches (49%), nose irritation (28%), and confusion (19%). In the office worker group, the most frequently complained symptoms were headaches (36%) and nose irritation (21%). The presence of subjective symptoms was significantly (*P* < .05) higher among nail workers than among the office workers, for example, headache, nausea, nose irritation, skin irritation, shortness of breath, and confusion. Although no cases were detected to exceed the VOEL, nine nail workers exceeded the VOEL after the Brief‐Scala model adjustment. As a result, nose irritation was significantly higher for nail workers who exceeded the VOEL of acetone.

Based on the review of chemicals in nail products, there is a variety of sources of each chemical. For example, acetone and xylene are mostly found in polish removers, butyl acetate, ethyl acetate, toluene, methyl ethyl ketone, and xylene are the components of solvents, diluent, adhesives, polish removers, and nail polishes. Vinyl acetate and benzene are two toxic chemicals used in nail polishes while methyl methacrylate and ethyl methacrylate typically used in artificial nails.^2^

We found that most of the chemicals in the nail salons had an impact on the respiratory and central nervous systems and caused skin irritation. Acetone, ethanol, butyl acetate, methyl ethyl ketone, and toluene can cause headaches and nausea. Ethyl acetate and toluene can cause confusion, and all chemicals can cause irritation, such as nose irritation and skin irritation. Shortness of breath is a primary symptom of methyl methacrylate and ethyl methacrylate exposure.^2^ The findings of the study suggest that nail workers exposed to a variety of chemicals have a higher proportion of symptoms than the comparison group. Moreover, the safety data sheet of acetone indicates that acetone cause irritation to the upper respiratory due to inhalation.^29^ Although the exposure limit in the profile of acetone is 750 ppm (OSHA), 250 ppm (NIOSH), and 500 ppm (ACGIH), which can impact the health of worker,^30^ the Vietnamese National Technical Regulation stated that lower exposure to acetone at 85 ppm (VOEL for acetone) cause health issues. According to the Ministry of Health, the VOEL needs to be adjusted for working hours if the worker spends more than 40 hours a week or 8 hours a day with the Brief‐Scala model.^31^ As a result, few workers in a salon exceeded the VOEL, while others in the same salon did not because they had longer working hours. Although the VOEL of acetone is significantly lower than OSHA or NIOSH, the findings show that if nail workers is near the higher limit of exposure, the nail workers tend to have a higher risk of nose irritation. The results of the study suggest that the nail workers should wear personal protective equipment to protect them from harmful chemicals in their workplaces such as charcoal masks, latex gloves, and glasses. In addition, employers can install local ventilation instead of general ventilation to help decrease the concentration of chemical exposure near the breathing zone.

Several factors can increase exposure to workers. Almost all the nail workers in our study were young adults with a mean age of approximately 24 years and a mean employment of 1.87 years. The nail industry in Vietnam is quite new and attracts many young females. However, since nail salon workers have long work days (mean 58 hours per week) they do have an extended period of time for exposure in general. Furthermore, there were two workers who lived in the workplace. We found the chemicals in the salon after their work shifts; hence, they were exposed to a higher concentration. We installed one sampler in the room where they stayed at night, and we recognize that they were still exposed to lower concentrations of VOCs. This means that the workers in their workplace may be exposed to higher concentrations than the visitors because the chemicals remain in the room if has a low air change rate.

Based on the Bayesian Model Averaging for choosing the best model for determining the relationship between the related factors and the concentrations of target VOCs and acetone, it was determined that the greater the number of customers that visited the salon, the greater the concentrations of both target VOCs and acetone in the environment. Thus, it appears that personal exposure is primarily influenced by the number of customers served. This is quite similar to the findings of a Korean study, where a high number of customers was associated with increased exposure to the chemicals measured^1^ The air velocity in the salons should not be used to predict the concentration of target VOCs. Most of the previous studies highly recommend using changes in air rates rather than air velocity.^32^ Many salons appear to be poorly ventilated based on the high concentration of CO_2_.^8^, ^16^, ^33^ Specifically, the predicted model indicates that if CO_2_ increases by 10 ppm, the concentration of target VOCs and acetone can increase 10.0 times.^1^ The concentrations of toxic chemicals were higher in the salons with general ventilation systems than in those without these systems, similar to other findings.^1^, ^5^ The presence of general ventilation increases the concentration of the target VOCs more than 2.5 times. Park et. al (2014) explained that general ventilation, particularly a ceiling exhaust duct, may spread the chemicals throughout a worker's breathing zone, and they suggested that local exhaust ventilation is more efficient in controlling exposure levels for nail workers.

We measured the concentration at the end of August. However, salon owners reported a high number of customers from January to June. As a result, the average number of customers per day reported in the questionnaire by the nail salon workers was higher than the number of customers we observed on the day of sampling. Compared to the Korean study, the concentration in our study was lower for a variety of reasons. First, the samplers in Korea were collected only during the weekend, so the number of customers may be higher than the average number of customers during the week. Second, the number of customers serviced in one day in Korean salons (5‐9 customers) is also higher than the number serviced in Vietnamese salons (2.85 customers).^1^ The personal concentrations of VOCs in our study were also lower than those measured in the United States.^5^, ^34^, ^35^ This is mainly due to the better indoor air quality of salons in Vietnam (mean of CO_2_ is 940 ppm compared with 1100 ppm in the United States).^16^ Similar to several studies conducted previously, the most common chemicals detected in our study were acetone, butyl acetate, methyl ethyl ketone, and ethyl acetate.^1^, ^5^, ^32^, ^36^

Although we did not measure the area concentration, personal samplers in or near the breathing zone should improve the estimated occupational exposure.^32^ All measured values were much lower than the occupational limits set by the OSHA or Vietnamese Ministry of Health without adjusting for the reduction factor for each worker. For example, the level of toluene was very low with a mean value of 0.09 ppm, similar to that reported in some studies in the United States.^37^ Some countries have replaced toluene with safer alternatives. In Vietnam, most of the nail products are imported from other countries, so the lower level of toluene can be understood. Long‐term exposure to benzene can cause cancer.^38^ The detection frequency of benzene is very low (21.4%) but similar to that reported in the United States (18%).^32^

The main limitation of the study was the low participation rate of salons (15 salons participated from 83 salons approached). The low participation rate may indicate a biased selection of salons, but the response rate of nail workers mitigated the low salon participation rate. On the other hand, due to the limited scope of the laboratory, we were not able to analyze all the chemicals in the passive samplers. Although we evaluated 21 nail workers alone, not all the 100 workers, the stratification of workers into SEGs allow limited resources to be allocated well. Thus, the total exposure present in a particular workplace can be characterized and managed effectively and efficiently.^39^ We did not measure the air quality in the office worker group's workplace, but some research indicated that the concentration of VOCs in the office building is significantly lower than the concentration among nail shops.^40^ We used office workers working in the offices adjacent to the salon as the comparison group to demonstrate that nail workers are at a higher risk of some symptoms than office workers. Although the nail workers exceeding VOEL had a higher risk of nose irritation, the relationship between chemical exposure and health outcomes should be conducted in prospective studies.

## 5 CONCLUSION

In conclusion, many hazardous chemicals are used in nail salons, such as acetone, that can lead to occupational symptoms among workers as opposed to in office workers. The study found that the target VOCs had a positive relationship with the number of serviced customers, concentration of CO_2_, general ventilation; acetone was the most frequently reported chemical with the highest concentration in nail salons, showing a positive relationship with the number of customers in salons, serviced customers, concentration of CO_2_, and air conditioner used. Finally, shop owners should improve their working conditions to protect their employees’ health, such as local exhaust ventilation. Nail workers should wear personal protective equipment to prevent occupational symptoms if engineering control is not available.

## CONFLICT OF INTEREST

*Approval of the research protocol:* The study was approved by the Human Ethical Committee of Thammasat University, Thailand (No.077/2562), and the Medical Ethics Committee Board of Danang University of Medical Technology and Pharmacy, Vietnam (No.01/2019/DHKTYDĐN). *Informed Consent:* All owners and nail workers were given information about the study and were asked to sign a consent form prior to their participation. *Registry and the registration no. of the study:* N/A*. Conflict of interest:* The authors declare no conflict of interest for this study.
